# Assessment of Prevalence of Adolescent Patient Portal Account Access by Guardians

**DOI:** 10.1001/jamanetworkopen.2021.24733

**Published:** 2021-09-16

**Authors:** Wui Ip, Samuel Yang, Jacob Parker, Austin Powell, James Xie, Keith Morse, Rachael C. Aikens, Jennifer Lee, Manjot Gill, Shravani Vundavalli, Yungui Huang, Jeannie Huang, Jonathan H. Chen, Jeffrey Hoffman, Cynthia Kuelbs, Natalie Pageler

**Affiliations:** 1 Department of Pediatrics, Stanford University School of Medicine, Palo Alto, California; 2 Information Services, Stanford Children's Health, Palo Alto, California; 3 Division of Clinical Informatics, Nationwide Children's Hospital, Columbus, Ohio; 4 Department of Hospital Medicine, The Ohio State University College of Medicine, Columbus; 5 Information Management Division, Rady Children's Hospital, San Diego, California; 6 Department of Anesthesiology, Perioperative and Pain Medicine, Stanford University School of Medicine, Palo Alto, California; 7 Department of Biomedical Informatics, Stanford University School of Medicine, Palo Alto, California; 8 Department of Pediatrics, The Ohio State University College of Medicine, Columbus; 9 Division of Pediatric Gastroenterology, Hepatology, and Nutrition, Nationwide Children's Hospital, Columbus, Ohio; 10 Department of Biomedical Informatics, The Ohio State University College of Medicine, Columbus; 11 Abigail Wexner Research Institute at Nationwide Children's Hospital, Columbus, Ohio; 12 Department of Pediatrics, University of California San Diego, La Jolla; 13 Center for Biomedical Informatics Research, Stanford University, Palo Alto, California; 14 Department of Medicine, Stanford University School of Medicine, Palo Alto, California

## Abstract

**Importance:**

Patient portals can be configured to allow confidential communication for adolescents’ sensitive health care information. Guardian access of adolescent patient portal accounts could compromise adolescents’ confidentiality.

**Objective:**

To estimate the prevalence of guardian access to adolescent patient portals at 3 academic children’s hospitals.

**Design, Setting, and Participants:**

A cross-sectional study to estimate the prevalence of guardian access to adolescent patient portal accounts was conducted at 3 academic children’s hospitals. Adolescent patients (aged 13-18 years) with access to their patient portal account with at least 1 outbound message from their portal during the study period were included. A rule-based natural language processing algorithm was used to analyze all portal messages from June 1, 2014, to February 28, 2020, and identify any message sent by guardians. The sensitivity and specificity of the algorithm at each institution was estimated through manual review of a stratified subsample of patient accounts. The overall proportion of accounts with guardian access was estimated after correcting for the sensitivity and specificity of the natural language processing algorithm.

**Exposures:**

Use of patient portal.

**Main Outcome and Measures:**

Percentage of adolescent portal accounts indicating guardian access.

**Results:**

A total of 3429 eligible adolescent accounts containing 25 642 messages across 3 institutions were analyzed. A total of 1797 adolescents (52%) were female and mean (SD) age was 15.6 (1.6) years. The percentage of adolescent portal accounts with apparent guardian access ranged from 52% to 57% across the 3 institutions. After correcting for the sensitivity and specificity of the algorithm based on manual review of 200 accounts per institution, an estimated 64% (95% CI, 59%-69%) to 76% (95% CI, 73%-88%) of accounts with outbound messages were accessed by guardians across the 3 institutions.

**Conclusions and Relevance:**

In this study, more than half of adolescent accounts with outbound messages were estimated to have been accessed by guardians at least once. These findings have implications for health systems intending to rely on separate adolescent accounts to protect adolescent confidentiality.

## Key Points

**Question:**

How frequently are adolescent patient portal accounts accessed by guardians?

**Findings:**

In this cross-sectional study including 3429 adolescent accounts across 3 academic institutions, analysis of portal messages found that more than half of adolescent patient portal accounts with outbound messages were accessed by guardians. The percentage of accessed accounts was greater in children aged 13 to 14 years vs those aged 17 to 18 years.

**Meaning:**

These findings may be useful in guiding health system approaches to protecting adolescent confidentiality when sharing health data via patient portals.

## Teaser

This cross-sectional study uses a natural language processing algorithm to estimate prevalence of access of adolescent patient portal accounts by guardians at 3 academic children's hospitals.

## Introduction

In many health systems, adolescents are permitted separate access to their electronic health record through an online patient portal. The adolescent account often supports appointment scheduling, record sharing, and communication with health care clinicians as a way to promote self-management and engagement as adolescents transition into adulthood.^1,2^ Confidential communication is necessary for many adolescents to feel comfortable seeking care for sensitive health needs (eg, pregnancy, sexually transmitted diseases, substance use).^3,4,5,6^ Furthermore, all 50 states in the US have some form of minor consent laws,^7^ which have associated rights to privacy protection for the minor according to the Health Insurance Portability and Accountability Act.^8^ Many health systems offer proxy portal accounts for legal guardians with separate login credentials to access selected portions of the adolescent portal accounts and communicate with their adolescents’ clinicians separately. However, there is evidence that guardians may be messaging directly from the patient account instead of using the intended proxy account. One study in adults with diabetes estimated that close to half of patient portal accounts have messages authored by proxies.^9^ However, the prevalence of this activity in adolescent patient portals is not known.

This issue is important in light of the 21st Century Cures Act Final Rule,^10,11^ which requires health systems to afford patients and their legal representatives access to selected portions of their health record electronically. If guardians use the adolescent’s account for portal access instead of their proxy account, sensitive information meant to be accessible only by the adolescent could become visible to others, thus compromising adolescent confidentiality.^8^ The objective of this study was to estimate the prevalence of guardian access of adolescent portal accounts by analyzing electronic messages across 3 academic children’s hospitals using a natural language processing (NLP) algorithm.

## Methods

This was a multisite cross-sectional study that estimated the prevalence of guardian access to adolescent patient portal accounts. Participating sites included 3 academic children’s health care institutions: Stanford Children’s Health (Palo Alto, California), Rady Children’s Hospital (San Diego, California), and Nationwide Children’s Hospital (Columbus, Ohio). At all 3 institutions, adolescents are permitted to have their own patient portal accounts and guardians can register for proxy accounts. All 3 sites use the same patient portal vendor (MyChart, Epic Systems Corp). Additional background information for the 3 institutions can be found in the eAppendix in the Supplement. The retrospective study was approved by the institutional review boards at all 3 institutions with waivers of informed consent. No identifiable data were shared among the 3 institutions. This study followed the Strengthening the Reporting of Observational Studies in Epidemiology (STROBE) reporting guideline.

We included adolescents aged 13 to 18 years with an active portal account and at least 1 outbound message between June 1, 2014, and February 28, 2020. We defined an outbound message as a message being sent from the patient portal to the health system. We excluded patients whose portal account had no outbound messages.

We developed a rule-based NLP algorithm to analyze all outbound messages from the adolescent portal accounts to identify messages that could reasonably be assumed to be authored by guardians. The algorithm flagged messages if the message contained any of the following features: (1) a third-person reference to the adolescent; (2) phrases such as my son, my daughter, or my child; or (3) the signature matched the name of a guardian on file. We subsequently identified the portal accounts associated with these flagged messages.

We defined an adolescent account with presumed guardian access (NLP-flagged account) if the account had at least 1 message flagged by the NLP algorithm. This NLP approach is a surrogate of true guardian access because some guardians may access adolescent accounts without messaging. The NLP algorithm was written in Python, version 3.7.7 (Python Software Foundation).

### Statistical Analysis

The performance of the NLP algorithm was validated through manual review of a sample of 200 accounts from each institution. To reduce sampling variability, messages were selected using a stratified random sampling scheme, with strata determined based on quartiles of the number of messages in each account. The number of accounts sampled from each stratum was determined using proportional allocation, so that the resulting sample was representative of all eligible accounts at the institution.^12^ Within each institution, 2 physician reviewers (J.X. and K.M., S.Y. and J.L, J.H. and C.K.) examined outbound messages associated with each account to independently annotate the presence of guardian access (manually flagged account). The reviewers were blinded to the algorithm outputs to reduce potential sources of bias. Interrater reliability was calculated using κ statistics.^13^ For any disagreements, the 2 reviewers discussed the annotations and reached a consensus. Using the manual annotation results as the reference standard, we calculated the sensitivity and specificity of the NLP algorithm for identifying accounts with guardian access.

After validation, we calculated performance-corrected estimates of the true number of accounts with guardian access at all 3 institutions (eMethods in the Supplement).^14^ Estimation of 95% CIs was performed with the superpopulation bootstrap for stratified samples^15,16^ followed by bias correction.^17,18^

As an exploratory analysis, using the subset of accounts with the reference standard annotations, we manually screened for any sensitive health topics mentioned in the messages. We defined sensitive health topics as services that adolescents can independently consent to and receive without permission from their guardians, including birth control, pregnancy, treatment for sexually transmitted diseases, substance use, and mental health problems.^7,19^ Between the 2 groups of accounts (with and without guardian access) using the manual validation scheme based on stratified random sampling, we compared the proportion of accounts with at least 1 adolescent message containing sensitive health topics by calculating the odds ratio.

## Results

We analyzed a total of 3429 eligible adolescent accounts containing 25 642 messages across 3 institutions. A total of 1632 adolescents (48%) were male and 1797 (52%) were female; mean (SD) age was 15.6 (1.6) years. Table 1 presents the number of NLP-flagged adolescent accounts (ie, accounts with ≥1 flagged message): 544 of 949 (57%) at Nationwide Children’s Hospital, 948 of 1780 (53%) at Rady Children’s Hospital, and 364 of 700 (52%) at Stanford Children’s Health. After adjusting for sensitivity (72%-81%) and specificity (91%-97%) for the NLP algorithm based on manual annotation results (200 accounts per institution), the estimated prevalence of guardian access of adolescent portal accounts was 64% (95% CI, 59%-69%) at Rady Children’s Hospital, 70% (95% CI, 62%-74%) at Stanford Children’s Health, and 76% (95% CI, 73%-88%) at Nationwide Children’s Hospital (Table 2). Sensitivity and specificity of the NLP algorithm for each stratum based on message count can be found in eTable 1 in the Supplement.

**Table 1.** Summary Statistics for Adolescent Patient Accounts

Variable	No. (%)		
	Nationwide Children’s Hospital	Rady Children’s Hospital	Stanford Children’s Health
Total adolescent accounts	1313	5452	2312
Eligible accounts (with ≥1 outbound message)	949 (72)	1780 (33)	700 (30)
Proxy account registered			
Yes	67 (7)	5 (0.3)	67 (10)
No	882 (93)	1775 (99.7)	633 (90)
Outbound messages			
Total	6364	13 841	5437
Flagged by NLP	2691 (42)	3941 (28)	1646 (30)
NLP-flagged accounts (accounts with ≥1 NLP-flagged message)	544 (57)	948 (53)	364 (52)
Age at the time of message, median (IQR), y	15.2 (14.1-16.2)	15.8 (14.8-16.9)	15.5 (14.3-16.4)
Sex			
Female	340 (36)	791 (44)	397 (57)
Male	609 (64)	989 (56)	303 (43)
Race and ethnicity^a^			
Asian/Pacific Islander	21 (2)	104 (6)	119 (17)
Black/African American	114 (12)	38 (2)	4 (.6)
Hispanic	30 (3)	441 (25)	94 (13)
Native American	1 (0.1)	0	2 (.3)
Other^b^	62 (7)	281 (16)	223 (32)
White Non-Hispanic	721 (76)	916 (51)	258 (37)

Abbreviations: IQR, interquartile range; NLP, natural language processing.

^a^ Based on self-reported data documented in the electronic health record.

^b^ Other refers to 1 of these 3 categories documented in the electronic health record: unknown, declined to state, or other.

**Table 2.** The Sensitivity and Specificity of the NLP Algorithm at Each Institution Using Manual Annotation as Reference Standard^a^

Variable	% (95% CI)		
	Nationwide Children’s Hospital	Rady Children’s Hospital	Stanford Children’s Health
NLP algorithm			
Sensitivity	72 (64-79)	81 (74-87)	73 (65-79)
Specificity	91 (80-97)	97 (88-100)	95 (85-99)
Estimated % of accounts with guardian access, after corrected for algorithm performance	76 (73-88)	64 (59-69)	70 (62-74)
κ Statistics for interrater reliability in manual review	0.83	0.81	0.93

Abbreviation: NLP, natural language processing.

^a^ The estimated prevalence of guardian access to adolescent portal accounts after correction for algorithm performance is listed.

The Figure shows that the percentage of NLP-flagged accounts had a general decrease as patients aged, from 59% to 64% in the 13- to less than 14-year age group down to 40% to 50% in the 17- to less than 18-year group. The eFigure in the Supplement shows no clear increase or decrease in the percentage of NLP-flagged accounts by calendar year across the study period. In addition, eTable 2 in the Supplement reports the top 10 departments at each institution based on the number of patient portal messages received. Our exploratory analysis also indicated that flagged accounts by manual review were less likely to have adolescent messages with sensitive topics at both Stanford Children’s Health (odds ratio, 0.05; 95% CI, 0.01-0.43) and Rady Children’s Hospital (odds ratio, 0.23; 95% CI, 0.07-0.83) (eTable 3 in the Supplement).

**Figure.**
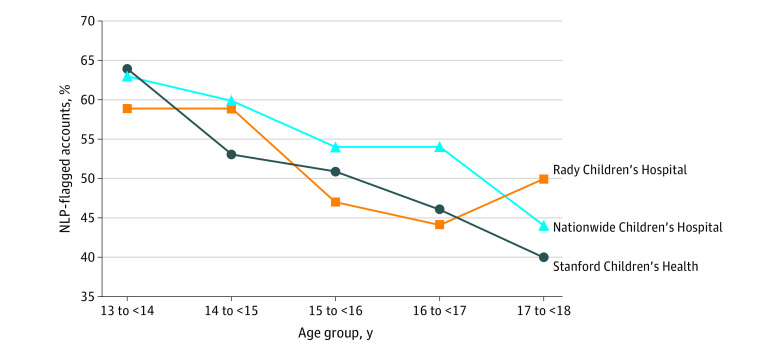
The Percentage of Natural Language Processing (NLP)–Flagged Accounts in Each Age Group

## Discussion

This multisite study provides an estimated prevalence of guardian access to adolescent patient portal accounts by analyzing electronic messages using an NLP algorithm. In our analysis, the estimated prevalence ranged from 64% to 76% across the 3 academic institutions, with a general decrease as patients aged. We believe this was the first study to evaluate the prevalence of guardian access of adolescent patient portal accounts.

Compliance with federal regulations, such as the 21st Century Cures Act, and state-specific consent and confidentiality laws for adolescents requires a reliable mechanism to share protected health information with adolescents without guardian knowledge. Pediatric institutions often rely on separate adolescent portal accounts with differential information access for this purpose, as recommended by several national organizations.^20,21,22^ Our study suggests that this practice may be insufficient to fully protect adolescent confidentiality. We hypothesize several possible reasons for guardian access of adolescent portal accounts: (1) institutional workflow issues, including errors during portal sign-up^23^; (2) misunderstanding of portal account design by adolescents and their guardians; (3) adolescents voluntarily sharing their portal access; and (4) guardians coercively or surreptitiously accessing the adolescent’s account.

For pediatric patients, guardians are often the first to request access to a child’s electronic health record, even before the patient’s adolescent years. During the process of providing portal access to the guardian, staff could erroneously provide access to the patient portal accounts by mistake. In general, adolescents and guardians may not be aware of the difference between proxy accounts and adolescent accounts. Wolff et al^24^ suggested the term *proxy*, commonly used by many health systems to describe shared access to patient portals, may not be familiar to a broad population. Thus, the guardian may have no awareness that they are using their child’s account rather than their own proxy account. This observation is consistent with our data, which show less than one-tenth of adolescent accounts also have registered proxy accounts. Because of this finding, quality improvement efforts to address institutional workflow issues have been initiated at Stanford Children’s Health to ensure appropriate portal account creation for both adolescents and their guardians. At Rady Children’s Hospital, adjustments are being made to organizational workflows to ensure that most adolescent accounts have a corresponding proxy account, and a multimodal communication campaign encourages parents and guardians to create a proxy portal account.

Another possible reason for the high percentage of guardian access is that adolescents voluntarily share their portal access with their guardians, negating the guardians’ need for separate proxy accounts. In a survey study, a small cohort of adolescents with cancer and blood disorders expressed that they had no concerns about what their parents would see in the patient portal.^25^ However, a survey study by Miklin et al^26^ reported that only half of the adolescents were familiar with the function of the patient portal; in addition, the adolescents were not aware that they could obtain care for mental health, sexual health, substance use, and pregnancy without parental permission. Therefore, adolescents may not fully understand the consequences of sharing portal access with their guardians, particularly that their guardians would continue to have access to their accounts and any associated sensitive health information.

In contrast, some guardians might choose to disregard adolescents’ privacy concerns, potentially coercing adolescents or surreptitiously gaining access to the adolescent’s account.^27^ One study found that about one-third of parents had negative opinions about privacy practices in adolescent clinics and disagreed with adolescents having private information.^28^ A national survey of 1000 parents revealed that most parents favor parental access to adolescent records even though they also believed this would cause adolescents to withhold information from physicians.^29^

Regardless of the actual reason in each case, our finding that a substantial portion of adolescent patient portal accounts were used by guardians raises questions. As an exploratory analysis, we observed that accounts of adolescent patients that appeared to be used by guardians were associated with fewer sensitive health topics in their messages to clinicians in a limited manual review sample. Although further studies with more data and control for confounders are warranted, our preliminary observation raises questions whether adolescents may be less willing to share sensitive health topics via the portal when aware that their guardians have access to their portal accounts. Previous studies have suggested that adolescents may not seek care for sexual health if parental notification was mandated.^5,6^

Based on these findings, it may be useful for health care systems to examine the current use of adolescent patient portals by guardians and develop strategies to promote proper portal access. It is necessary to educate adolescents and their guardians on the concepts of patient portals and proxy accounts, as well as the benefits and limitations of electronic communications,^30^ especially given that many adolescents are not familiar with patient portals.^26^ Ramsey et al^31^ described the outcome of the use of dedicated staff to engage and educate adolescent patients about the portal in clinics, as inspired by customer service strategies used by large technology companies to promote technology adoption. Dedicated staff for portal sign-up could help ensure contact information associated with the account belongs to the adolescents instead of the guardians. Clinician training related to confidentiality within the electronic record is also necessary to protect sensitive information for adolescents.^32^ In addition, technological improvements are needed to enable differential access of clinical information between adolescents and guardians to protect adolescent confidentiality.^30,33^

### Limitations

The study has limitations. Although this study suggests that it is common for guardians to use their adolescents' accounts, it provides little insight into the underlying reasons for this behavior. Additional investigation is needed to better understand the perspectives of guardians and adolescents with regard to their use of patient portals to inform which strategies are best to ensure adolescent confidentiality. In addition, because our NLP algorithm is rule-based, it is less adaptive to the message context compared with a machine learning approach. For instance, if the patient’s name was listed as Susannah in the electronic health record but a message from a guardian referred to her as Sue, the rule-based NLP algorithm would not recognize the reference to the patient but a clinician reviewer likely would. This limitation could in part explain higher flagged numbers in manual review compared with the NLP algorithm. The rules in the NLP algorithm were designed to minimize false-positives, which can cause the algorithm to become less sensitive when the volume of account messages is low. Another limitation of our study is that guardian access was identified based only on message content. Guardians who do not send messages via their adolescents’ portal accounts might still be accessing these accounts to obtain sensitive information—a breach of confidentiality that is undetectable by analysis of messages alone. Nevertheless, the estimates reported in the study can be viewed as the lower bounds for guardian access during the study period as their true access rate, such as when messages are not sent, could be higher. Applying an NLP algorithm to portal messages could serve as a monitoring tool for guardian access of adolescent portals, adding a safeguard against potential compromise of adolescent confidentiality.

## Conclusions

Using an NLP algorithm to analyze adolescent portal messages, we estimate that more than half of adolescent portal accounts with outbound messages appear to have been accessed by guardians, which may pose challenges for health systems intending to rely on adolescent portals to protect adolescent confidentiality.

## Notes

Supplement. **eAppendix.** Background Information on the 3 Academic Institutions **eMethods.** Performance-Corrected Estimate of the True Proportion of Accounts With Guardian Access **eTable 1.** NLP-Algorithm Sensitivity and Specificity for Each Stratum Based on Message Counts **eTable 2.** Top 10 Departments at Each Institution Based on the Number of Adolescent Patient Portal Messages Received **eFigure.** The Percentage of NLP-Flagged Accounts Based on Calendar Year **eTable 3.** Comparing the Proportion of Accounts With at Least 1 Adolescent Message That Contains Sensitive Topics Between Flagged and Non-Flagged Accounts
