# Supplementary material for: Assessment of Prevalence of Adolescent Patient Portal Account Access by Guardians
Source: JAMA Netw Open. 2021 Sep 16;4(9):e2124733. doi: 10.1001/jamanetworkopen.2021.24733 (PMC8446820; doi:10.1001/jamanetworkopen.2021.24733)
Supplement: Supplement. — eAppendix. Background Information on the 3 Academic Institutions eMethods. Performance-Corrected Estimate of the True Proportion of Accounts With Guardian Access eTable 1. NLP-Algorithm Sensitivity and Specificity for Each Stratum Based on Message Counts eTable 2. Top 10 Departments at Each Institution Based on the Number of Adolescent Patient Portal Messages Received eFigure. The Percentage of NLP-Flagged Accounts Based on Calendar Year eTable 3. Comparing the Proportion of Accounts With at Least 1 Adolescent Message That Contains Sensitive Topics Between Flagged and Non-Flagged Accounts [file jamanetwopen-e2124733-s001.pdf]

Supplementary Online Content

Ip W, Yang S, Parker J, et al. Assessment of prevalence of adolescent patient portal account access by guardians. *JAMA Netw Open*. 2021;4(9):e2124733. doi:10.1001/jamanetworkopen.2021.24733

eAppendix. Background Information on the 3 Academic Institutions

eMethods. Performance-Corrected Estimate of the True Proportion of Accounts With Guardian Access

eTable 1. NLP-Algorithm Sensitivity and Specificity for Each Stratum Based on Message Counts

eTable 2. Top 10 Departments at Each Institution Based on the Number of Adolescent Patient Portal Messages Received

eFigure. The Percentage of NLP-Flagged Accounts Based on Calendar Year

eTable 3. Comparing the Proportion of Accounts With at Least 1 Adolescent Message That Contains Sensitive Topics Between Flagged and Non-Flagged Accounts

This supplementary material has been provided by the authors to give readers additional information about their work.

© 2021 Ip W et al. *JAMA Network Open.*

eAppendix: Background information on the 3 academic institutions

Nationwide Children's Hospital

Nationwide Children's Hospital is a free-standing tertiary children's hospital with 1.6 million patient visits in 2020 including 210,000 primary care visits and 431,000 specialty clinic visits. During the study period, Nationwide Children's Hospital (NCH) activated patient portals at parent or patient request, either during an in-person encounter or through an online request process. Some clinics (e.g. primary care, cardiology) undertook quality improvement projects during the study period, during which time patient portal activation was encouraged. Prior to 2016, activation codes were either emailed or printed. In 2016, NCH added the ability to send a text message with an activation link. Teenagers are able to request and activate their portal account without parental involvement. During the study period, adolescents were required to review and sign an acknowledgment form prior to issuing a proxy account to their guardian since, at the time, there was no difference in content or features between adolescent patient and proxy portal accounts.

Rady Children's Hospital

Rady Children's Hospital San Diego is a free-standing tertiary care children's hospital providing care to over 90 percent of the children in the San Diego region with over 1.2 million patient visits in 2020 including 500,000 primary care visits and 260,000 specialty visits. During the study period, parents and legal guardians of patients were encouraged to sign up for proxy patient portal accounts and given an activation code as part of the visit summary or discharge instructions at the conclusion of outpatient, inpatient, and emergency encounters. Adolescent patients are required to have parental consents on file in order to activate portal accounts. While proxy accounts are encouraged for parents and guardians of adolescents, organizational policies do not require an active proxy account in order for adolescents to have their own portal accounts.

Stanford Children's Health

Stanford Children's Health is a pediatric and obstetric tertiary health system that includes a 361-bed free-standing children's hospital and 65 ambulatory clinic locations providing care to the Northern California area and outreach to the western United States with an annual volume of 215,000 primary care visits and 325,000 specialty clinic visits. During the study period, adolescent patients and their legal guardians were encouraged to sign up for patient portal accounts and proxy accounts during any ambulatory clinic visits or after a hospitalization. An activation code was delivered through email, text or mail. Adolescent patients can activate patient portal accounts without parental consents, but the account functions only limit to provider messaging and viewing upcoming appointments. With parental consents, adolescents can activate more advanced features such as scheduling appointments and viewing laboratory results.

© 2021 Ip W et al. *JAMA Network Open.*

eMethods: Performance-corrected estimate of the true proportion of accounts with guardian access

If $\tilde{\theta}$ represents the proportion of NLP-flagged accounts at an institution, then the performance-corrected estimate of the true proportion of accounts with guardian access at that institution can be calculated by:

$$\hat{\theta} = \frac{\tilde{\theta} - (1 - sp)}{se + sp - 1}$$

where se and sp represent the sensitivity and specificity of the NLP algorithm estimated at the institution, respectively.

© 2021 Ip W et al. *JAMA Network Open.*

eTable 1: **NLP-algorithm sensitivity and specificity for each stratum based on message counts** (straum 1 = lowest number of messages per account; stratum 4 = highest number of messages per account)

	**Nationwide Children's**		**Rady Children's**		**Stanford Children's**	
	Sensitivity (%)	Specificity (%)	Sensitivity (%)	Specificity (%)	Sensitivity (%)	Specificity (%)
Stratum 1	55 (40-70)	100 (81-100)	59 (41-76)	97 (83-100)	50 (37-63)	93 (76-99)
Stratum 2	63 (45-78)	95 (76-100)	81 (65-91)	94 (70-100)	75 (54-88)	92 (62-100)
Stratum 3	82 (65-92)	82 (51-96)	80 (62-91)	100 (63-100)	85 (66-94)	100 (68-100)
Stratum 4	86 (72-94)	33 (6-80)	96 (84-100)	100 (51-100)	93 (79-98)	100 (63-100)

Footnotes (n = number of messages in an account):
Stratum 1 - NCH: n <= 1; RCH: n <= 1; SCH: n <= 2
Stratum 2 - NCH: 1 < n <= 3; RCH: 1 < n <= 3; SCH: 2 < n <= 4
Stratum 3 - NCH: 3 < n <=7; RCH: 3 < n <=7; SCH: 4 < n <= 10
Stratum 4 - NCH: n > 7; RCH: n > 7; SCH: n > 10

© 2021 Ip W et al. *JAMA Network Open.*

eTable 2: Top 10 departments at each institution based on the number of adolescent patient portal messages received

We use the recipient's departmental affiliation in the electronic health record at the time of the message (N = message count, Pt = patient count). The top 10 specialties account for 66%, 79%, and 69% of the messages at Nationwide Children's, Rady Children's and Stanford Children's respectively.

Nationwide Children's			Rady Children's			Stanford Children's		
**Specialty**	**N**	**Pt**	**Specialty**	**N**	**Pt**	**Specialty**	**N**	**Pt**
Gastroenterology	1342	159	Pediatrics	2869	676	Gastroenterology	884	90
Rheumatology	652	87	Endocrinology	1962	325	Rheumatology	501	46
Behavioral Health	403	90	Gastroenterology	1571	219	Pain Medicine	493	37
Psychiatry	390	118	Dermatology	1041	183	Psychiatry	421	52
Adolescent Medicine	376	90	Rheumatology	915	87	Endocrinology	312	76
Neurology	300	86	Neurology	750	125	Neurology	305	55
Endocrinology	239	75	Orthopedics	622	157	Dermatology	211	53
Pediatrics	195	75	Cardiology	437	74	Diabetes	211	47
Developmental Behavioral Pediatrics	164	17	Allergy	418	71	Pediatrics	205	85
Gynecology	154	39	Rehabilitation	364	47	Immune Behavioral Health	193	6

© 2021 Ip W et al. *JAMA Network Open.*

eFigure: The percentage of NLP-flagged accounts based on calendar year

If an account has at least one NLP-flagged message sent within a calendar year, the account is flagged in that year.[a]

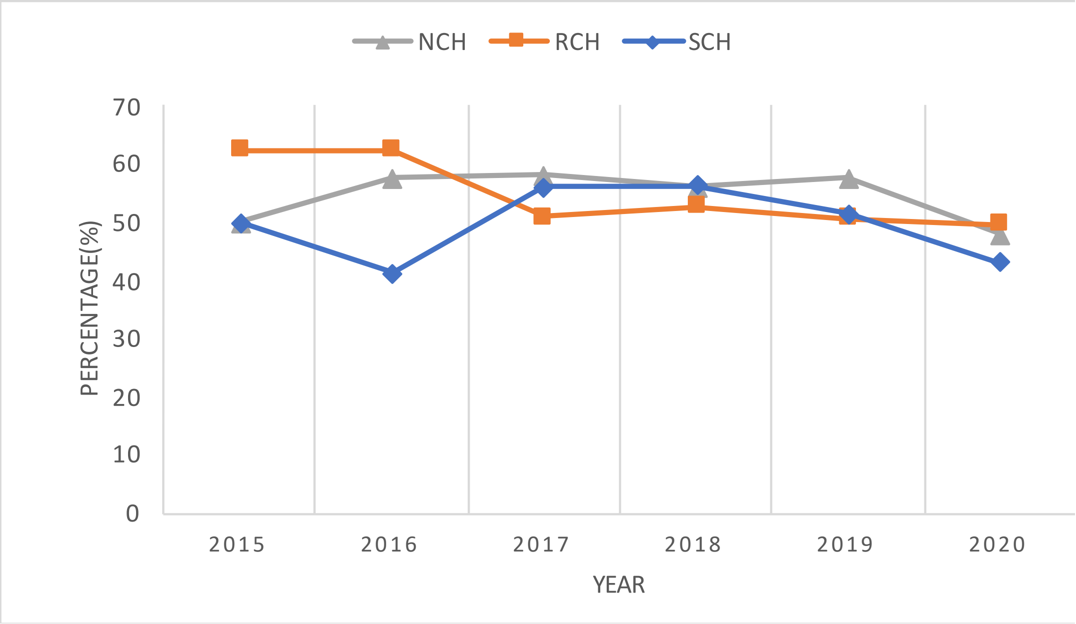

Footnote:

a. our earliest messages were found in 2015, despite our initial database query was set to June 2014 as our inclusion criteria

© 2021 Ip W et al. *JAMA Network Open.*

eTable 3: Comparing the proportion of accounts with at least 1 adolescent message that contains sensitive topics between flagged and non-flagged accounts. The comparison is based on manual review of the stratified random samples of 200 accounts at each institution

	Sensitive topics	Flagged accounts	Non-flagged accounts	OR (95% CI)
Nationwide Children's Hospital	Yes	13	10	0.46 (0.19, 1.11)
	No	131	46	
Rady Children's Hospital	Yes	4	7	0.23 (0.07, 0.83)
	No	134	55	
Stanford Children's Health	Yes	1	7	0.05 (0.01, 0.43)
	No	141	51	

© 2021 Ip W et al. *JAMA Network Open.*
